# Beyond the gut: Non‐specific interstitial pneumonia in pediatric ulcerative colitis—A rare extra‐intestinal manifestation

**DOI:** 10.1002/jpr3.70289

**Published:** 2026-11-05

**Authors:** Harshit Vachher, Vishrutha Poojari, Shivangi Tetarbe, Parmarth Chandane, Lakshmi Shobhavat, Ira Shah

**Affiliations:** ^1^ Department of Pediatric Gastroenterology and Hepatology Bai Jerbai Wadia Hospital for Children Mumbai Maharashtra India; ^2^ Department of Pediatric Respiratory Medicine Bai Jerbai Wadia Hospital for Children Mumbai Maharashtra India; ^3^ Department of Pediatric Critical Care Bai Jerbai Wadia Hospital for Children Mumbai Maharashtra India

**Keywords:** interstitial lung disease, macrophage activation syndrome, pediatric inflammatory bowel disease

## Abstract

Inflammatory bowel disease (IBD) is a chronic inflammatory disorder often presenting with extra‐intestinal manifestations (EIMs) involving the skin, joints and eyes. However, pulmonary manifestation, such as interstitial lung disease (ILD), is a rare complication, particularly in the pediatric population. We report a case of a 10‐year‐old boy, with very early onset IBD‐ulcerative colitis, presented with non‐productive cough, fever and dyspnea, with examination findings revealing respiratory distress, pallor, clubbing and pyoderma gangrenosum. High‐resolution computed tomography (HRCT) was suggestive of bilateral diffuse ground‐glass opacities with intralobular septal thickening and tractional bronchiectasis, consistent with non‐specific interstitial pneumonia (NSIP). Furthermore, the patient developed septic shock and macrophage activation syndrome, requiring intensive care management and immunosuppression. This case highlights that even in the setting of silent intestinal disease activity of a pediatric IBD case, the patient can have potentially life‐threatening pulmonary EIMs, warranting early diagnosis and treatment.

## 1 INTRODUCTION

Inflammatory bowel disease (IBD) is a broad entity that includes Crohn's disease (CD), ulcerative colitis (UC), and IBD‐unspecified. The Global Burden of Disease Study 2021 analyzed among children and adolescents (age 0–19 years) from 1990 to 2021, that the number of IBD cases increased by approximately 13.8%, climbing from 12,305.32 to 14,007.76.^1^ Despite this rise in absolute numbers, the incidence rate per 100,000 remained essentially unchanged, showing a marginal dip from 0.54 to 0.53, indicating an overall stable trend.^1^ In IBD, rectal bleeding is commonly associated with UC rather than CD, but patients with CD often have weight loss and perianal disease.^2^ IBD is a disease with multi‐systemic involvement; thus, patients often have extra‐intestinal manifestations (EIM). Approximately 28% of pediatric patients at the diagnosis of IBD have EIM.^3^ EIMs predominantly involve 4 systems: skin, joints, biliary tract, and eyes.^4^ Skin‐related EIMs include aphthous stomatitis, pyoderma gangrenosum, erythema nodosum and psoriasis.^4^ Other EIMs include peripheral arthritis, axial arthropathy, primary biliary cholangitis, pancreatitis and uveitis.^5^ EIMs are more commonly seen in CD compared to UC.^3^ Pulmonary manifestations are considered rare in IBD, which include interstitial pneumonitis, panbronchiolitis, bronchiolitis obliterans organizing pneumonia, pulmonary vasculitis, Langerhans cell histiocytosis and sarcoidosis.^6^ We present a 10‐year‐old boy on treatment for UC since 8 years with lung involvement in the form of non‐specific interstitial pneumonia (NSIP), which is a rare presentation.

## 2 CASE REPORT

A 10‐year‐old male child, second by birth order, born of a non‐consanguineous marriage, was diagnosed with very early‐onset IBD–UC at 2 years of age, in view of recurrent diarrhea with colonoscopic findings of left‐sided rectosigmoid ulcers and biopsy suggestive of focal mucosal ulceration with cryptitis and crypt abscess, and stool calprotectin of 234 mcg/g. Whole exome sequencing (WES) was normal. He had been treated with sulfasalazine and azathioprine since the age of 2 years. He had also received infliximab, three doses for multiple perianal fistulas at 2½ years of age, and later stopped in view of monetary constraints. Azathioprine was also stopped at 5 years of age in view of leukopenia and recurrent *Staphylococcus epidermidis* chronic suppurative otitis media (CSOM). At 9 years of age, in view of recurrent CSOM, paroxysmal cough, and wheezing, a pulmonary function test (PFT) was done, which showed a mild restrictive pattern. Diarrhea had been under remission since 4 years of age. He presented to us in September 2025 at 10 years of age with low‐grade fever, non‐productive cough, and breathing difficulty for 5 days. On examination, weight was 20.6 kg, and height was 128.5 cm, <3rd centile and 25th−50th centile, respectively, as per Indian Academy of Pediatrics (IAP) charts. He had a respiratory rate of 32 breaths per minute; heart rate was 128 beats per minute with blood pressure of 120/65 mm of Hg. There was pallor, clubbing, cervical lymphadenopathy, and pyoderma gangrenosum in the left leg (Figure 1). On respiratory system examination, there were bilateral basal crepitations; other systems were normal. Investigations revealed pancytopenia, raised erythrocyte sedimentation rate (ESR) and C‐reactive protein (CRP) (Table 1). Chest X‐ray showed bilateral ground‐glass opacities with fluffy shadows.

**Figure 1:**
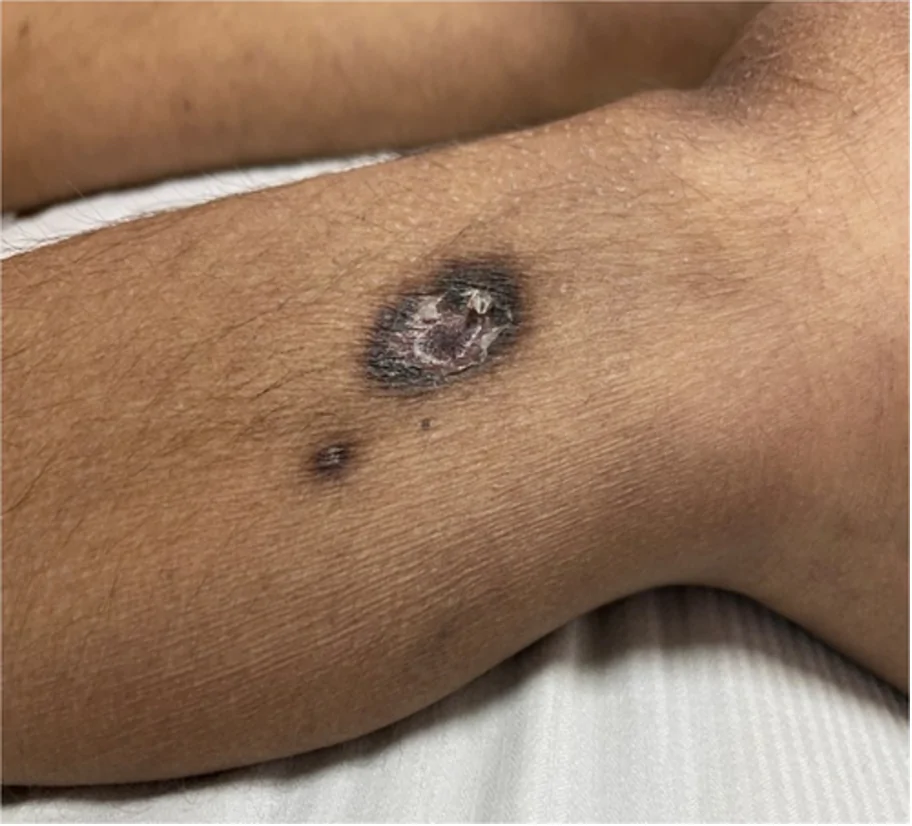
Pyoderma gangrenosum on left lower limb.

**Table 1:** Serial investigations of the patient.

Investigations	Day 1 of hospitalization	Day 7 of hospitalization	Day 13 of hospitalization	Day 30 of hospitalization	On discharge	On follow‐up
Hemoglobin (g/dL)	6.6	8.7	8.2	7.8	8.6	10.2
TLC (mm^3^)	6100	4500	600	5590	8030	7080
ANC (mm^3^)	4800	3870	230	4080	6022	5734
Platelets (mm^3^)	86,000	113,000	6000	21,000	42,000	99,000
Sr. sodium (mmol/L)	140	131	133	137	133	140
Sr. potassium (mmol/L)	3.9	4.4	4.3	3.6	4.9	4.2
Sr. chloride (mmol/L)	108	97	103	100	97	103
Sr. calcium (mg/dL)	8.3	8.9	9.2	8.1	8.9	9.2
Sr. creatinine (mg/dL)	0.29	0.23	0.18	0.22	0.2	0.36
SGOT (U/L)	32	132	2637	55	41	29
SGPT (U/L)	7	157	241	37	26	18
Total bilirubin (mg/dL)	0.8	1.2	2.4	2.1	1.3	0.8
Direct bilirubin (mg/dL)	0.2	0.5	2.1	1.9	1.0	0.2
Sr. albumin (g/dL)			1.9	4.1	3.8	3.8
PT (s)	11.7	15	11.8	11.3	11.4	10.2
INR	0.95	1.23	0.96	0.91	0.93	0.82
CRP (mg/dL)	36	13.5	20.4	10	1.3	0.6
ESR (mm in the first hour)	137		82	42	18	16
Ferritin (ng/mL)			51,175	3917		
Triglycerides (mg/dL)			285	294		
Fibrinogen (mg/mL)			63.9	104.9		
Interleukin‐6 (pg/mL)			11.55			

Abbreviations: ANC, absolute neutrophil count; CRP, C‐reactive protein; ESR, erythrocyte sedimentation rate; INR, international normalized ratio; PT, prothrombin time; SGOT, serum glutamate oxaloacetate transaminase; SGPT, serum glutamate pyruvate transaminase; Sr., serum; TLC, total leukocyte count.

High‐resolution computed tomography (HRCT) chest showed diffuse patches of ground‐glass opacities with intralobular septal thickening and tractional bronchiectasis of the bilateral upper lobes of the lungs, along with necrotic changes in hilar nodes suggestive of NSIP (Figure 2). Pediatric ulcerative colitis activity index (PUCAI) was 25, denoting mild disease activity. Broncho‐alveolar lavage (BAL) Xpert mycobacterium tuberculosis/rifampicin assay, fungal culture, and respiratory multiplex polymerase chain reaction panel were negative. BAL galactomannan was positive with an index of 0.79 (normal <0.50). He was put on non‐invasive ventilation and started on intravenous (IV) liposomal amphotericin‐B; cefosulbactam and sulfasalazine were stopped. Despite ongoing treatment, he had persistent fever spikes and developed septic shock 13 days after hospitalization, with cultures being negative, necessitating inotropic support, which was complicated by development of hepatosplenomegaly and macrophage activation syndrome (Table 1), for which intravenous immunoglobulin (IVIG) at 2 gm/kg and dexamethasone at 10 mg/m^2^ for a duration of 10 days were given. He also received multiple settings of transfusion of blood products along with granulocyte colony‐stimulating factor (G‐CSF). In view of lack of clinical and biochemical improvement following corticosteroid therapy, and as biological therapy was not feasible due to financial constraints, pulse IV cyclophosphamide (500 mg/m^2^) was started. He gradually improved, and oral prednisolone (2 mg/kg/day) was started; on Day 40 of hospitalization, he was discharged on monthly IV cyclophosphamide for 6 months with trimethoprim‐sulfamethoxazole prophylaxis. On follow‐up after 1 month for the next IV cyclophosphamide dose, the child had weight gain with improvement in lab parameters (Table 1). Four months into treatment, the child again developed high‐grade fever and breathing difficulty for 3 days, which did not respond to medical management, and subsequently he succumbed to illness.

**Figure 2:**
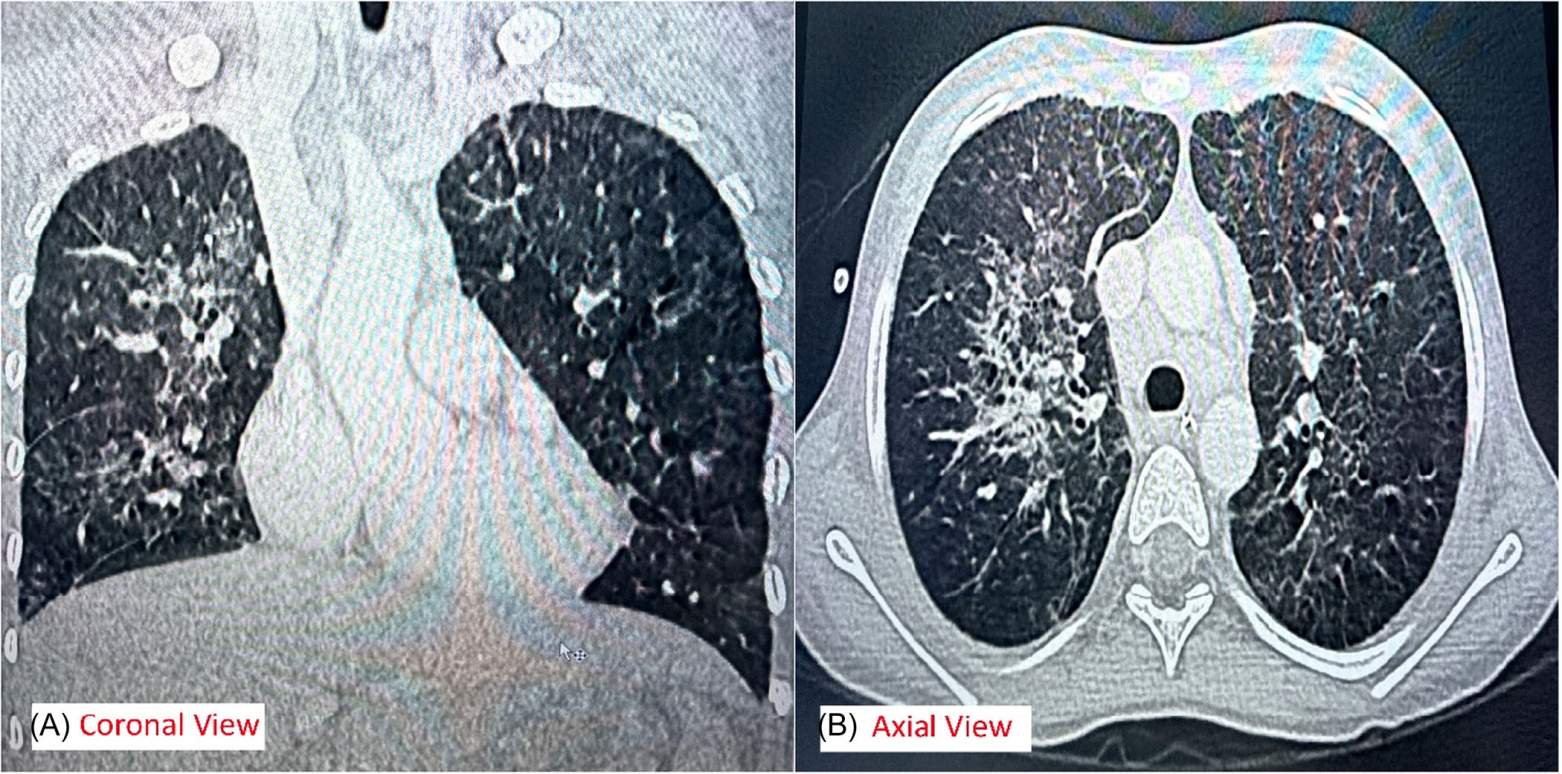
CT chest showing non‐specific interstitial pneumonia. (A) Coronal view. (B) Axial view. CT, computed tomography.

## 3 DISCUSSION

UC is a disease chiefly involving the colonic mucosa, but it can manifest with extra‐intestinal complications. Among these, pulmonary involvement is rare in children, as stated in a study conducted in Sweden between 1969 and 2019; the incidence rate was found to be 34 cases per 10,000 person‐years.^7^ The proposed pathogenesis includes the shared embryologic derivation of the intestinal and respiratory tracts, dysregulation of immune responses, and aberrant inflammatory signaling beyond the gut mucosa. Pulmonary manifestations may arise at any time, unrelated to intestinal disease activity. HRCT is the imaging modality of choice, capable of detecting parenchymal changes such as ground‐glass opacities, reticulations, and septal thickening. PFTs show a restrictive pattern and reduced diffusing capacity. Therapeutically, there is no standardized protocol due to the rarity of such cases. Though corticosteroids remain first‐line with variable responsiveness, in refractory or severe disease, immunomodulators like azathioprine, methotrexate, or biologic agents may be considered.^8^ This pediatric case highlights the necessity for vigilance: unexplained respiratory symptoms in a child with UC merit thorough evaluation. A multidisciplinary team encompassing gastroenterologists, pulmonologists, and radiologists is vital to ensure timely diagnosis and to guide individualized therapy.

## 4 CONCLUSION

Although pulmonary complications in pediatric IBD are rare, they can represent a serious EIM. Interstitial lung disease (ILD) in this setting, while uncommon, may carry significant morbidity. Early diagnosis through careful clinical evaluation, HRCT imaging, and lung function testing is crucial. Initiation of corticosteroids or appropriate immunosuppression at an early stage may improve outcome and minimize chronic lung damage. This report underscores that UC‐associated ILD should be included in the differential when a child with IBD develops unexplained respiratory symptoms.

## CONFLICT OF INTEREST STATEMENT

The authors declare no conflicts of interest.

## ETHICS STATEMENT

Written informed consent was obtained from the patient's guardians for publication of this case report and images.
